# Healthcare professionals’ views on healthcare-related factors influencing symptom course in persistent somatic symptoms: a qualitative study of four European countries

**DOI:** 10.1186/s12913-025-12986-1

**Published:** 2025-06-11

**Authors:** Aleksandra Kustra-Mulder, Brodie McGhie-Fraser, Tara Petzke, Karolina Fila-Pawłowska, Judith Rosmalen, Fiammetta Cosci, Bernd Löwe, Angelika Weigel

**Affiliations:** 1 Department of Psychosomatic Medicine and Psychotherapy, University Medical Center Hamburg-Eppendorf, Hamburg, Germany; 2 Department of Primary and Community Care, Radboud University Medical Center, Research Institute for Medical Innovation, Nijmegen, Netherlands; 3 Department of Clinical Psychology, Psychotherapy, and Experimental Psychopathology, Johannes-Gutenberg-University Mainz, Mainz, Germany; 4 Department of Clinical Neuroscience, Wrocław University of Science and Technology, Wrocław, Poland; 5 Departments of Psychiatry and Internal Medicine, University of Groningen, University Medical Center Groningen, Groningen, the Netherlands; 6 Department of Health Sciences, University of Florence, Florence, Italy

**Keywords:** Persistent somatic symptoms, Functional disorders, Healthcare, Diagnosis, Treatment, Psychotherapy

## Abstract

**Background:**

The care trajectory for patients with Persistent Somatic Symptoms (PSS) is complex due to variability in diagnoses and treatments, with differences across European healthcare systems. Existing findings predominantly come from individual Western European countries, and comparative studies are lacking. This study aimed to explore how healthcare systems are perceived to influence PSS courses across four European countries and how professionals view their respective systems regarding PSS.

**Methods:**

We used semi-structured interviews to conduct a qualitative study with healthcare professionals from Germany, Italy, the Netherlands, and Poland. Sixteen participants were recruited purposively through international and national networks focusing on PSS, ensuring representation from primary care, secondary care medical specialists, mental health, and other healthcare fields.

**Results:**

We found that the interaction of structural and interpersonal factors within the healthcare system influenced the course of PSS symptoms. Systemic barriers such as limited consultation times and issues in care pathways or insurance coverage were prevalent in Germany and the Netherlands, while access and trust issues were more prominent in Italy and Poland. Key improvements suggested included reimbursement and treatment eligibility for PSS, establishing collaborative care pathways, and sufficient consultation times. Additionally, enhancing professional-patient relationships and improving education for healthcare professionals and patients were identified as crucial steps.

**Conclusions:**

The results show that although expertise is improving, current healthcare system structures prevent professionals from using them effectively. Therefore, systemic reforms and better professional training are needed to improve care for patients with PSS.

**Supplementary Information:**

The online version contains supplementary material available at 10.1186/s12913-025-12986-1.

## Background

Persistent Somatic Symptoms (PSS) are subjectively distressing physical complaints that persist on most days for several months or more, regardless of their cause [1, 2]. These symptoms can be a part of somatic illness, functional somatic disorders, mental health conditions, or undiagnosed conditions. PSS are commonly encountered, occurring in more than 50% of outpatient consultations across various settings [3]. PSS may present as single symptoms, such as pain, dizziness, fatigue, or gastrointestinal issues, or multiple symptoms [4]. PSS can cause substantial individual burden [5], and may be accompanied by comorbid somatic and mental disorders [2, 6].

Affected individuals often struggle to obtain an appropriate diagnosis and treatment, frequently having to seek care from various healthcare professionals [7, 8]. PSS can be part of a wide range of conditions and are therefore classified under many diagnostic labels. Various treatments are offered to patients depending on their point of contact within the healthcare system. The variability in diagnoses and treatments adds to the challenge for healthcare professionals, who frequently face time constraints, have limited training in managing such conditions, and must navigate diverse healthcare services that may lack a unified approach to PSS [9]. These challenges have been reported to contribute to feelings of anxiety, frustration, and a self-perceived lack of competency. Responses to this have included over-investigation or avoidance of patient contact [10].

Previous research has shed some light on PSS healthcare organizations across Europe [11], and the high healthcare costs associated with PSS [12]. However, this research has been predominantly from Western Europe, and studies comparing European healthcare systems are lacking. Further, little is known about how facets of various healthcare systems might influence the course of PSS.

Healthcare systems differ widely across Europe [13] in terms of care aspects, such as the function of general practitioners (GPs) as gatekeepers, the extent to which healthcare services are refunded by statutory or additional private insurance, and availability of guidelines for treating PSS [13, 14]. Additionally, there are differences in healthcare provision within each country, which may include regional variations depending on the specific illness being addressed. Andersen’s behavioral model of healthcare utilization, widely applied in various areas of health care and illness, offers a theoretical framework to account for these differences [15, 16]. A recent scoping review applied Andersen’s model to investigate the influence of systemic factors, such as the organization of care, health policies, or insurance coverage, on the course of PSS across Europe [17]. The review found that the treatment setting was the only identifiable factor due to the insufficient description of care provided in the included studies. Additionally, the included studies predominantly came from the United Kingdom, Germany, and the Netherlands. Therefore, further research is needed to explore other systemic factors within the model that may be influencing the course of PSS and to understand these dynamics across different countries.

Healthcare professionals’ perspectives are vital to gaining in-depth insights into the systemic factors, the care structure and organizations for PSS, and their potential impact on the course of illness. A recent survey with healthcare professionals across Germany, Italy, the Netherlands, and Poland provided an important first step regarding the identification of healthcare-related aspects of PSS associated with symptom persistence, deterioration, and improvements [18]. Healthcare professionals across the investigated countries agreed that multidisciplinary collaboration, particularly the integration of psychological and medical care, would facilitate PSS improvement. When asked about the systemic aspects related to symptoms persistence or deterioration, healthcare professionals differed in their responses. Professionals in Italy and Poland highlighted issues related to access and availability of care, while those in Germany and the Netherlands focused on the content and implementation of care. However, the brief responses to the open-ended questions limited the depth of insight into healthcare-related aspects that could be targeted to develop concrete strategies to improve the PSS symptom course across Europe. Thus, further qualitative research was crucial to delve deeper into the nuances of healthcare provision for PSS.

This qualitative study aimed to explore (1) how aspects of the care system are perceived to affect the course of symptoms in PSS (persistence, deterioration, or improvement) across various European countries and (2) how healthcare professionals perceive their respective healthcare systems concerning PSS.

## Methods

This study is part of ETUDE (Encompassing Training in Functional Disorders Across Europe; https://etude-itn.eu/), an innovative training network aiming to improve the understanding of the mechanisms, diagnosis, treatment, and stigmatization of PSS [19]. Healthcare professionals from Germany, Italy, the Netherlands, and Poland with experience in caring for patients with PSS were eligible to participate in a semi-structured interview. The included countries were chosen to represent Europe’s northern, southern, western, and eastern regions and the ETUDE network. The exclusion criteria were a lack of experience working with patients with PSS and insufficient verbal communication in German, Italian, Dutch, or Polish. The COREQ guideline was used for the reporting of this study [20].

The participants gave their verbal informed consent before the interviews. As this study neither involved vulnerable groups nor any forms of interventions, there were no specific ethical issues to consider. Therefore, the study did not require ethical approval as it did not fall under paragraph 6 of the Helsinki Declaration, the guidelines of the Ethics Committee of the Hamburg Medical Association in Germany, the Medical Research Involving Human Subjects Act (WMO) in the Netherlands, Law No. 3 of January 11, 2018, Ministerial Decree of January 30, 2023, Regional Law No. 40/2005 in Italy, or the guidelines of the Bioethics Committee at the Medical University of Wroclaw in Poland. All methods followed relevant guidelines and regulations, including the Declaration of Helsinki. The study was pre-registered at the Open Science Framework registry (OSF: https://osf.io/dp5fj).

### Participants

Participants were recruited via email between August 2022 and January 2023 using contacts from the ETUDE and EURONET-SOMA networks [4, 19] and national networks. A purposive sampling strategy was adopted in each country, aiming to include one representative from primary care (e.g., GP), a secondary care medical specialist, a mental health specialist, and another healthcare professional such as a physiotherapist or nutritionist. This aimed to ensure representation across different treatment settings for PSS and a wide spectrum of experience in diagnosing and treating patients with PSS. The semi-structured interviews were conducted in person to explore the participants’ views of the healthcare system’s potential influence on the course of PSS and their perception of health care provided in their respective countries. A semi-structured interview guide was developed for this study and translated into all four languages (the English version is available on the OSF: https://osf.io/j63s4/).

The interviews were conducted by an interdisciplinary and international research team (see Supplementary Material 1 for researcher characteristics). All interviews were conducted in the practitioners’ work environments, such as clinics or hospitals, except for one interview in Poland, which was conducted at the practitioner’s home due to the rural location of their practice. No relationship with the participants was established before the study’s commencement. However, the participants were informed about the study aims and received a brief overview of the interviewers’ research background.

### Qualitative data analysis

Interviews were audiotaped and transcribed verbatim, assisted by an online transcription and editing platform (www.trint.com). The qualitative analysis was conducted within the native languages to prevent the loss of language- and culture-specific content [21]. The coding and codes were applied in English, which was the common language within the group of coders. Thematic analysis was chosen as a flexible yet structured approach to coding the qualitative analysis in English while leaving the interviews in their native language and allowing for both deductive and inductive theme identification [22, 23]. Two coders per country, with at least one native in the relevant language and the others being proficient, coded the interviews (see Supplementary Material 2 for the codebook).

First, all coders familiarized themselves with the data by listening to the interviews and reading the transcripts, in line with the initial phase of thematic analysis [22]. This step helped ensure a shared understanding of the material across different languages and coders. While no formal coding was conducted at this stage, reflections and early impressions that emerged during familiarization were discussed within the team and later considered during inductive coding. To provide a structured starting point, initial codes were then developed deductively, based on the research questions and Andersen’s model [15], and later complemented by inductive codes that emerged through deeper engagement with the data. We adopted a critical realist standpoint as our ontological stance, acknowledging that reality is socially influenced and only partially accessible. Despite differences in country and professional background, healthcare professionals’ perceptions of reality were sufficiently congruent with one another to gain meaningful insights [24]. This approach was relevant to our research because we asked healthcare professionals to discuss the health systems in which they worked and how these affected the patients being treated in them. Healthcare professionals’ insights were based on their specific knowledge of the local health system and their interactions with patients. We adopted contextualism, an epistemological paradigm, which posits that reality and its perception are always context-bound [24], guiding our interpretation of participants’ accounts within their specific healthcare settings.

Through an iterative process and recurring online discussions between the coding group, codes were redefined and condensed to themes as meaningful units. Any disagreements were resolved in discussion with the study group. The developed themes were then recursively reviewed by referring back to the original material from each country. Similarities and differences between countries were reflected on, as were the researchers’ pre-assumptions and interpretations. These revised themes were defined and labeled, with quotes from each country identified for illustration. Lastly, relevant themes were structured and reported to answer the research questions. MAXQDA software was used for qualitative analysis (Release 23.0.0, VERBI GmbH, 2023).

Besides co-coding and regular discussions within the coding team, further techniques to enhance trustworthiness and intersubjective consistency in the analysis included member checking, where the coders assessed the accuracy of researchers’ interpretations and discussed the findings in relation to the existing literature.

## Results

### Participant characteristics

In total 16 healthcare professionals were recruited, with four from each country. Healthcare professionals included one GP, one mental health specialist (i.e., psychotherapist or psychiatrist), one secondary care medical specialist (i.e., internist, gynecologist, rehabilitation specialist), and one other healthcare professional (i.e., physiotherapist or nutritionist) per country, respectively. They included 10 female participants (62%). The age range was 31 to 66 years (M = 48.4 years, SD = 11.4) and work experience ranged from 3 to 39 years (M = 16.8 years, SD = 9.7). Interviews lasted on average 49 min (range: 37–81 min).

### Main themes and subthemes

**Fig. 1:**
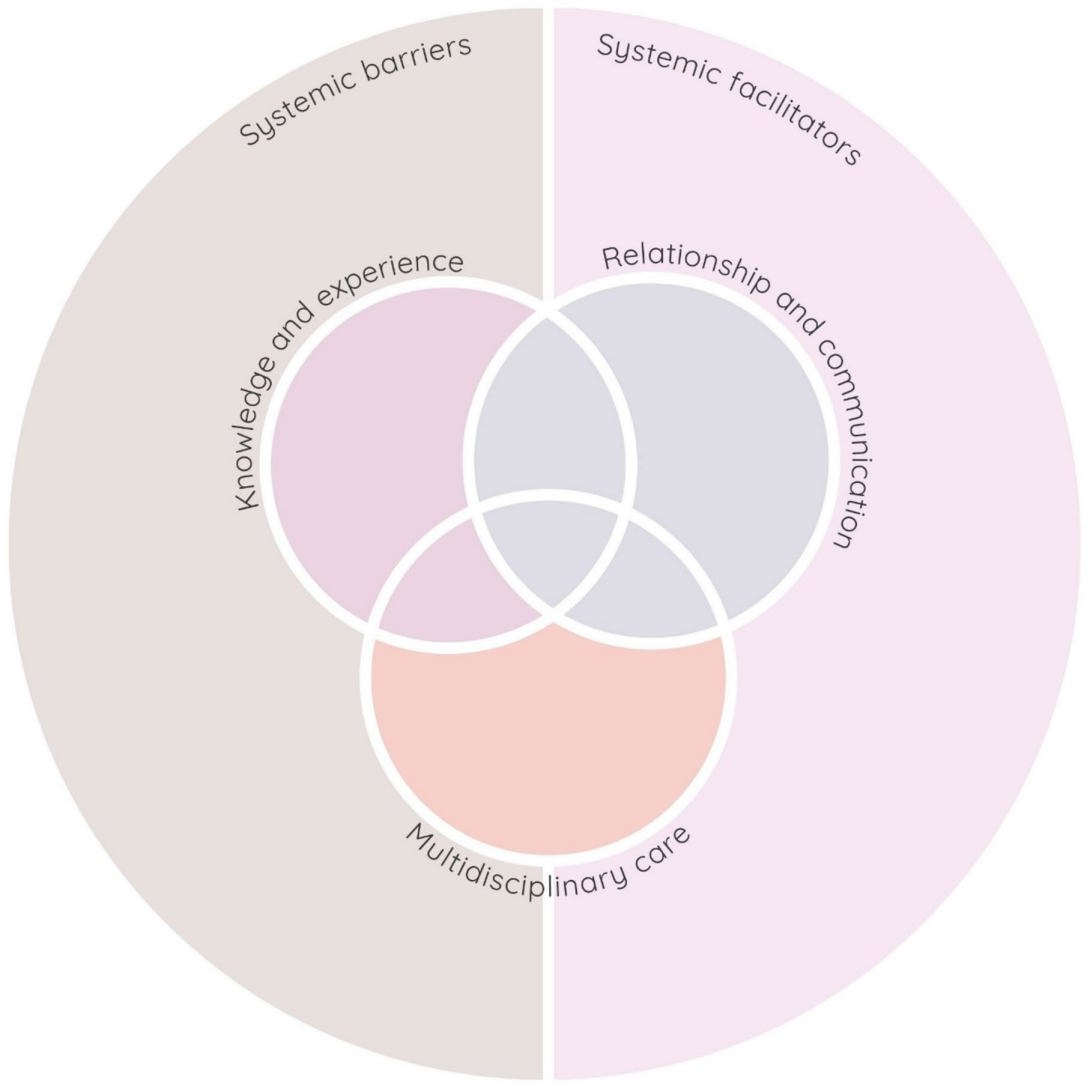
Themes identified in interviews with healthcare professionals from four European countries

**Fig. 2:**
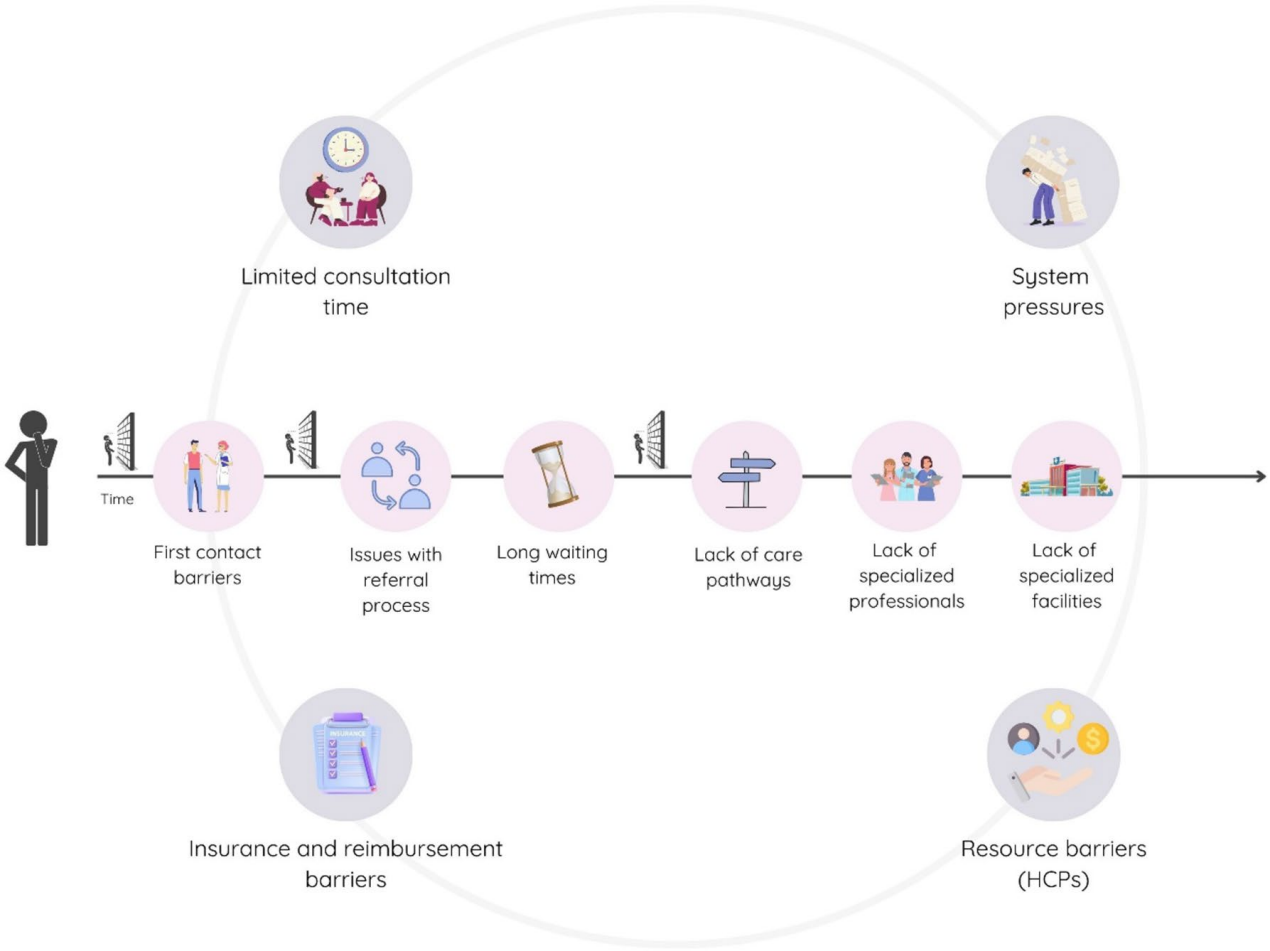
Examples of common systemic barriers (Theme 1) for PSS care across four countries

Qualitative results indicated that the PSS symptom course was influenced by the interaction of systemic and interpersonal factors of the healthcare system. At the systemic level, both (1) systemic barriers and (2) systemic facilitators shaped the trajectory and type of care for patients provided within each healthcare system. At the interpersonal level between healthcare professionals and patients, we found that several domains directly affected the quality of the interaction and ongoing relationship. These were: (3) multidisciplinary care, (4) healthcare professionals’ knowledge and experience of PSS, and (5) relationship and communication. These interpersonal themes were found to to both reflect barriers and facilitators. Figure 1 gives an overview of the five main themes. To illustrate the impact of systemic factors on the PSS symptom course, we have contextualized potential barriers described within a patient’s care journey. Figure 2 presents examples of these barriers, throughout the patient’s care journey, depicting the progression from the initial consultation to the ongoing management and treatment stages.

#### Theme 1. Systemic barriers

Many barriers that take place during the patient care trajectory were mentioned, highlighting the complexity of challenges faced by patients and healthcare professionals and the heterogeneity within healthcare systems. Figure 2 illustrates common systemic barriers across countries as part of Theme 1. Although these similarities existed in the perceived barriers, participants in Germany and the Netherlands focused primarily on barriers related to the structure of the existing healthcare system, such as insurance and reimbursement issues, the organization and implementation of care pathways, and the need for better coordination of follow-up care. In contrast, participants in Italy and Poland highlighted barriers to availability and practical access to care, including levels of trust and structural limitations within their healthcare systems, such as a lack of dedicated pathways, guidelines, and insufficient specialist professionals.

##### Subtheme 1.1. Systemic barriers along the care trajectory

Throughout the care trajectory, the barriers mentioned were mainly related to the first contact, referral process, and access to specialist pathways (see Fig. 2).

Several systemic issues were highlighted regarding the patient’s first contact, typically within primary care. The role and perception of GPs varied significantly across different healthcare systems, affecting patient care. In the Netherlands, the barrier was described on an individual level, with healthcare professionals noting that some GPs lacked the necessary affinity and competency in managing PSS, creating early obstacles for patients seeking appropriate care. In contrast, in Poland, the problem was regarded as systemic, with GPs described as lacking authority and consequently not being the first point of contact for patients. Polish participants described a prevailing image that GPs were not competent or qualified to treat PSS, which would lead patients to bypass primary care and seek specialist care directly through private clinics at their own expense. This was perceived as a perpetuated cycle of mistrust and delayed care.

*“There’s also this stereotype perpetuated that GPs somehow don’t have the competence and qualifications to treat psychosomatic symptoms or symptoms under the somatic form… patients don’t trust GPs in this area*,* especially those* [patients] *who have persistent somatic symptoms…*,* and therefore they turn to specialists*,* going through a long queue of consultations*,* often very lengthy.”* (Psychiatrist, Poland).

After the initial consultations, participants described a heavy reliance on the referral process. Dutch professionals suggested that GPs’ limited familiarity and clarity concerning PSS referrals might frequently lead to excessive or insufficient referrals, initiating a cycle of ineffective communication that could prolong patient suffering because symptoms would persist without resolution.

*“But then*,* uh*,* yeah*,* then you quickly get*,* uh*,* this back and forth*,* so to speak*,* with the patient coming back again*,* and then they see me again and say*,* “Well*,* I’ve been to a specialist*,* nothing came out of it*,* but I still have symptoms”*,* and then it starts. Actually*,* nothing has changed.”* (GP, the Netherlands).

Systemic issues were also seen as hindering the referral process. In Italy, the process was described as further complicated by difficulties in accessing psychological consultations, with no straightforward way to request them.

*“We have a lot of psychology services that are difficult to reach*,* psychiatry equally difficult. (…) If you think you can request a consult with them*,* as you would request a consult for a cardiovascular or cardiac condition from a cardiology service*,* this is not possible. There is no such thing or at least in our reality it does not exist.”* (GP, Italy).

German professionals noted specific structural limitations that created obstacles within their referral system, including the ineligibility of PSS for certain treatments such as physiotherapy.

After referral to specialized care, long waiting times were emphasized across all countries. These were described as ranging from several months to a couple of years, with particular delays in mental health care, and were identified as a relevant reason for illnesses to be increasingly severe and more difficult to treat.

*“… And they* [the patients] *wait like that for a couple of months*,* half a year*,* where there’s just such a progression of the illness*,* and sometimes it’s too late to reverse the cause.”* (Physiotherapist, Poland).

Long waiting times were also noted to be further exacerbated by reluctance of staff to accept patients with PSS with mild symptoms or complex conditions due to concerns about increasing their workload and operating beyond their clinical expertise.

*“… Psychologists find pain quite complicated… there are hardly any psychologists who say*,* well*,* I want to deal with pain.”* (Rehabilitation specialist, the Netherlands).

These delays were also regarded to lead to out-of-pocket expenses and patients opting to see specialists privately, especially in countries with private clinics such as Italy and Poland. Healthcare professionals noted that– if available - patients with sufficient financial resources would perceive private clinics as more attractive than public care to evade long waiting times.

*“At the private level certainly the delivery of a service is faster than*,* than in the public service.”* (Nutritionist, Italy).

*“There is certain difficulty*,* certain roughness*,* discomfort with public healthcare*,* that if everything went smoother there*,* maybe patients would think less about financing treatment from their own funds.”* (Psychiatrist, Poland).

Struggles with referrals and long waiting times were further attributed to a perceived scarcity of healthcare professionals with expertise in managing PSS. This shortage would lead to challenges in finding and receiving appropriate care, resulting in inadequate treatment or the overlooking of patients’ conditions.

*“Nowadays*,* patients are sometimes sent to me specifically because there are not so many specialized options for people with chronic complaints*,* they don’t get as much support in their practice*,* in the outpatient clinic outside*,* so to speak.”* (Gynecologist, Germany).

Healthcare professionals in Germany and Italy also noted systemic barriers to care accessibility, including the absence of specialized facilities dedicated to PSS. This was particularly the case in rural areas.

*“I believe that in rural regions*,* there is significant under-provision*,* not only in the hospital sector but also in the outpatient sector for somatic issues. And for psychosomatic competence or linked psychosomatic competence*,* one could certainly create a large shortage map.”* (Gynecologist, Germany).

The lack of specialized care facilities was described to lead to disjointed patient experiences, resulting in patients having to navigate their care without clear treatment plans. Professionals indicated that such gaps resulted in patients consulting multiple physicians in search of relief, with little coordination or oversight, unless the patient’s health significantly deteriorated. The system was failing both patients and healthcare professionals, as healthcare professionals would be overwhelmed, and patients would not receive adequate care:

*“The problem is always the usual one*, i.e.,* it revolves around the fact that since there are no dedicated pathways the accessibility is poor and so… what often happens is that these patients um (reflecting) wander a bit aimlessly*,* if you can call it that*,* and so they don’t find their place* [in terms of specialized care], *they don’t come i.e./or the intake is done very late.”* (Internist, Italy).

##### Subtheme 1.2. Overarching systemic barriers

In addition to the stage-specific ones, broader systemic barriers, such as limited consultation times, system pressures, resource barriers for healthcare professionals, and insurance coverage, could significantly affect patient care throughout the healthcare trajectory. A representation of these barriers is presented in Fig. 2.

Limited consultation times were a commonly mentioned obstacle to quality patient care. Healthcare professionals described balancing extensive patient care with strict schedule constraints.

*“I think the problem*,* as I understand it from patients*,* is often a lack of time. When patients actually want to say a lot more than the doctor is perhaps signaling that they want to listen. We have time pressure in general practice*,* especially when there are a lot of acute patients in addition to the appointment consultation hours…”* (GP, Germany).

In the Netherlands, managing patients with PSS was described as challenging due to time constraints and limited reimbursement for inter-professional consultations (e.g., with GPs or psychologists), which often had to take place outside regular working hours or were skipped entirely. This was seen as compromising the quality of care.

System pressures, such as high patient volumes, administrative tasks, and difficulties coordinating follow-up care, were reported to contribute to the challenge in providing quality care. Polish participants pointed out that healthcare professionals had to deal with high patient loads and a ‘factory work’ healthcare environment, forcing them to rely on prescribing medication and quick advice due to time constraints.

*“However*,* in the clinic*,* sometimes the number of patients is so large that in order not to sit at work until evening*,* I have to shorten the consultation time a bit. It’s frustrating*,* but unfortunately*,* that’s how it looks*,* that’s reality.”* (Psychiatrist, Poland).

Resource barriers for healthcare professionals also made it difficult to provide quality care. These included a lack of institutional support and resources to access other specialists or consultations for their patients, low hourly rates for certain services, and insufficient funding, particularly in specialized areas like psychiatry. Furthermore, healthcare professionals expressed ambivalence between the wish to provide care for as many patients as possible and the constraints of limited time per patient.

*“Clearly*,* when you’re taking on patients*,* you naturally want to provide care for them. But the more patients you accept*,* the less time remains for each individual. That needs to be taken into account and should also be communicated externally. And then*,* if necessary*,* it results in a stop to new admissions. It’s certainly tough when someone with psychosomatic complaints is seeking a consultation and is not accepted as a new patient.”* (GP, Germany).

Beyond consultation time constraints, insurance coverage and reimbursement presented additional systemic barriers to quality care. In the Netherlands, healthcare providers were described as increasingly recognizing the biopsychosocial model, but treatment frameworks and insurance systems remained rooted in traditional medical approaches. This resulted in gaps in care for patients with PSS.

*“…But our treatment frameworks are still focused on musculoskeletal complaints*,* while our knowledge is increasingly about*,* you know*,* the functional level or the brain and the stress system. And with that*,* we see that we could actually treat people with SOLK* [medically unexplained symptoms] *very well in these settings because they are dealing with the underlying patterns. But because of our own treatment frameworks and also because of the Health Insurance Act*,* we are not allowed to treat those patients.”* (Rehabilitation Specialist, the Netherlands).

Insurance coverage was described to be coupled with strict limitations of insurance policies. These would often cover only specific treatments or diagnoses and fail to consider the psychological components of these conditions. In the Netherlands, psychosomatic physiotherapy would only be covered by supplementary insurance, which some people could not or were unwilling to afford. Additionally, due to the lack of recognition of PSS as a chronic condition in the Dutch insurance system, patients with these conditions do not qualify for extended coverage that would allow for longer treatment duration. Consequently, patients’ only option would be to rely on supplementary insurance, which they often could not afford. In Italy, specific costs of PSS treatment were described as not being covered by insurance schemes. If a patient had a comorbidity like cancer, in select cases they might receive PSS treatment as part of their overall care. This would create a financial disincentive for practitioners due to inadequate reimbursement for the extensive diagnostic requirements of PSS care.

*“These are disorders that pay little* [to us doctors] *compared to the many diagnostic investigations.”* (Internist, Italy).

In Germany, statutory health insurance made healthcare professionals hesitant in prescribing certain treatments due to budget constraints and concerns about exceeding insurance limits. On the other hand, German healthcare professionals stressed that patients with private insurance faced a higher risk of overdiagnosis as financial incentives for doctors might encourage excessive use of diagnostic procedures.

*“In patients with private insurance*,* I often notice*,* much to my chagrin*,* that there is so much diagnostics going on and there is really a lot of doctor hopping. Not only from the patient’s side*,* but also “Do this and that again and have this checked out again”. Although the last MRI really wasn’t that long ago. From my point of view*,* this has to do with the insurance status.”* (Psychologist, Germany).

#### Theme 2. Systemic facilitators

While systemic barriers were the predominant focus in the healthcare professionals’ responses, a few examples of systems facilitators were provided that could improve the symptom course for patients with PSS. In addition, hopes and ideas were presented for future systems improvements.

##### Subtheme 2.1. Effective practices

Similar to the barriers presented, respondents also outlined certain examples of practices considered effective within their country’s healthcare systems. While German and Dutch professionals highlighted national initiatives and policies, Italian and Polish professionals referred more to effective practices implemented at specific clinics or local settings, with Italy also reflecting aspirations for broader care integration.

In the Netherlands, these included a national initiative called “Meer tijd voor de patiënt” (More time for the patient), which extended consultation times for complex cases, particularly those with persistent complaints. The aim was to provide more comprehensive care by allowing doctors to delve deeper into the various factors affecting a patient’s symptoms and experiences. Other practices included accessible primary care with short waiting times, and specialized centers offering multidisciplinary and comprehensive care with pre-consultation assessments. In Germany, identified successful strategies included special billing codes within statutory health insurance to reimburse practitioners for longer consultations with complex cases, flat-rate compensation model at outpatient university clinics, curricula fostering multidisciplinary communication, and treatment intermissions to evaluate patient self-management.

*“Yes*,* we are now being compensated a bit better for consultation time. In the statutory health insurance system*,* there are certain billing codes*,* like a consultation code*,* and there is also a psychosomatic code; these cover about 10–15 minutes. Now*,* as part of the general practitioner program*,* most contracts include this in the flat rate*,* and it is better compensated.”* (GP, Germany).

In Poland, an interviewee working in a specialized rehabilitation center described their practice as having no time constraints for consultations, enabling access to professionals and round-the-clock nursing staff. While the interviewee viewed the holistic approach and extended patient stays as effective, these practices were setting-specific and not directly related to PSS alone. In Italy, healthcare professionals shared current practices and aspirations for improving the management of PSS. Effective practices included collaborations with psychologists, providing psychological guidance and support alongside medical treatment, and fostering a strong professional-patient relationship through empathetic listening and patient empowerment in decision-making.

##### Subtheme 2.2. Desired changes, ideas, and solutions

Healthcare professionals voiced a common desire for systemic transformation to improve patient care. Practitioners across countries stressed the need to reduce waiting times to ensure timely and effective care. In Italy and Poland, there was a push for dedicated clinics with shorter waiting times, and for reducing waiting times during the initial diagnostic phase to provide more timely care. In Germany, the concept of treating patients within medical care centers (MVZ) was highlighted, to allow patients in specialized medical care more consultation time through additional billing codes, comparable to the GP setting.

Clearer and more efficient treatment pathways were a common request. In the Netherlands, there was a desire for a better referral process and a more comprehensive basic insurance plan that includes coverage of psychosomatic physiotherapy. German practitioners noted the need for mechanisms that would facilitate access to physiotherapy, such as a “blank prescription” option, which would allow patients to seek physiotherapy as needed and not bound to a certain number of sessions, which needed to be scheduled within a certain time frame. In Italy, creating dedicated treatment pathways was seen as crucial for managing patient care effectively.

#### Theme 3. Multidisciplinary care

Participants consistently highlighted the benefits of multidisciplinary care for patients with PSS. A collaborative, interdisciplinary, team-based approach was perceived to provide comprehensive and effective treatment. Professionals emphasized that close collaborations among specialists from various disciplines could enhance treatment planning, deepen the understanding of patient complaints, and reduce unnecessary procedures, such as excessive imaging.

*“…And the facilitators*,* it can be a multidisciplinary team. So the interaction between different professionals who are trained and obviously also possibly a dedicated treatment trajectory um [reflecting] with consultants; when the trajectory exists they can be called to be consulted*,* perhaps by the doctor who*,* who may have this hunch*,* but not the experience and skills to recognize it promptly.”* (Nutritionist, Italy).

Several challenges were identified in implementing interdisciplinary collaboration. Professionals from Germany noted that contradicting opinions among healthcare providers regarding diagnostic procedures and treatment options impeded the establishment of cohesive care. Additionally, the lack of secure infrastructure for collaborative treatment, such as digital solutions or regular interdisciplinary meetings, hindered interdisciplinary care in Germany.

*“But if I were to say now that we have patients in our outpatient practice and we want to hold consultation again*,* that would be very difficult. And I think a platform*,* something like a board or a digital exchange platform*,* which is protected and where those involved can actually exchange information*,* that’s what’s missing.”* (Physiotherapist, Germany).

Italian professionals mentioned that the lack of integrated treatment pathways made collaboration challenging, as there was no dedicated treatment trajectory to facilitate collaboration. Patients seeking multiple opinions from medical doctors further complicated care schedules and treatment plans.

Effective care coordination was also noted as crucial for improving outcomes for patients with PSS. Across countries, the role of the GP as a central coordinator was emphasized, guiding patients through the healthcare system and ensuring seamless communication among various healthcare providers. However, this role may not always be effectively fulfilled due to structural issues, such as lack of time, resources, and financial incentives. The absence of a coordinating figure in the care pathway was regarded to lead to patients being trapped in repetitive treatment cycles or unnecessary consultations.

*“This causes patients to find themselves in closed treatment loops from which they cannot escape. So*,* the lack of a doctor who could guide the treatment and whom patients could trust is the main reason for ineffective treatment*,* sometimes unnecessary consultations*,* or hospital stays.”* (Psychiatrist, Poland).

Amid the challenges to multidisciplinary care, the importance of integrating psychological support was consistently mentioned across countries. Healthcare professionals advocated for better integration of psychological care to improve symptom management and help patients recognize the role of psychological factors in their conditions.

*“This link to or between somatic functional and psychiatric. So*,* I believe the psychiatric is again a whole different world in itself*,* and we are not yet so used to dealing with it in an interdisciplinary way. So*,* it is completely separated*,* psychiatric and somatic. And*,* uh*,* yes*,* that there is also no development in that area according to my perception.”* (Gynecologist, Germany).

#### Theme 4. Healthcare professionals knowledge and experience

Healthcare professionals across all countries noted varying levels of knowledge and understanding of PSS. These were often still rooted in a biomedical model, which affected care delivery. Despite exposure to PSS within different medical specializations, education in this area was described as remaining insufficient, highlighting a systemic gap in medical curricula.

*“Sure*,* in theory*,* it was covered. From today’s perspective or from the perspective of the early years of work*,* I found that it wasn’t enough to… get a good handle on dealing with the patients*,* and also to*,* well*,* to get a good approach there. I didn’t feel well-trained in that regard.”* (Psychotherapist, Germany).

Personal encounters with patients with PSS were indicated to be crucial for healthcare professionals to fill these educational gaps, leading to divergent responses to the lack of formal education. Some healthcare professionals recognized their knowledge gaps and used this awareness to engage in self-directed learning and professional development.

*“At work*,* I was helpless in the face of these disorders*,* the symptoms*,* and the patients. I lacked a language for communicating with patients and an idea of how to treat this disorder. So*,* one could say that I have educated myself in this area.”* (Psychiatrist, Poland).

However, opposite reactions of healthcare professionals were also mentioned: after personal encounters with patients with PSS, some professionals reported having become disengaged or developed negative stereotypes towards patients with PSS, adversely affecting care quality.

*“They are also more complex and time-consuming to begin with*,* and they might get on your nerves at some point because they take resources away from others.”* (Physiotherapist, Germany).

Healthcare professionals saw a positive shift toward a biopsychosocial perspective, indicating a move toward a more comprehensive and holistic approach to healthcare. This perspective was described as being gradually implemented in interdisciplinary training and considering multiple aspects of health and behavior.

*“Rehabilitation is inherently the domain of interdisciplinary treatment*,* with a biopsychosocial perspective on health and behavior. So*,* in that training*,* we are broadly educated to take a broad approach and think broadly. Uh*,* which means that chronic musculoskeletal pain is also part of that training. I also see a shift in this area in the last 20 years.”* (Rehabilitation specialist, the Netherlands).

There was consensus that current education needed improvement through enhanced training opportunities and continuous professional development. Healthcare professionals posited that building strong foundational expertise would be essential because it could influence healthcare providers’ interactions with patients and affect care at various stages.

A key conflict in healthcare emerged in the interaction between the growing skills of dedicated professionals and the constraints of healthcare systems that would often impede the practical implementation of such expertise.

#### Theme 5. Relationship and communication

A strong professional-patient relationship was consistently mentioned as crucial in managing patients with PSS, influencing treatment outcomes and patient satisfaction. Good relationships were regarded to be built on empathetic communication, patient engagement, and a thorough understanding of the patient’s context. Healthcare professionals noted that when patients felt heard and understood, it allowed providers to identify appropriate intervention points.

*“The contact with the patient is very important and I think that applies to both mild and severe PSS*,* right. So when a patient really feels heard and understood*,* uhm*,* then you more easily get to the core of the matter*,* I think*,* with that patient… the moment a patient feels understood and heard*,* they will open up. And then uh*,* well then I think a GP will more easily find the right points of intervention.”* (Psychotherapist, the Netherlands).

In contrast, a lack of clear communication or dismissiveness led to confusion and dissatisfaction. Professionals emphasized taking complaints seriously and understanding the patient’s condition as important steps toward effective care. Additionally, avoiding nocebo language—negative suggestions that could exacerbate symptoms—along with anxiety and visible discomfort from the professional was considered important, as it could hinder progress. Instead, communication focused on a biopsychosocial approach would support patients’ understanding and acceptance of their conditions.

However, professionals noted a need for improvement in this area. Systemic issues, such as constraints on healthcare budgeting in Germany, or staff overloads and administrative burdens in Poland, posed significant challenges to nurturing the professional-patient relationships.

*“I think the system causes that an overworked doctor*,* nurse*,* social worker*,* psychologist*,* psychiatrist*,* anyone who is overwhelmed with formalities*,* above all*,* not even by the patients themselves*,* but by the formalities*,* to be overwhelmed and unable to approach the patient with genuine interest because they themselves are simply tired*,* exhausted.”* (Physiotherapist, Poland).

The importance of high-quality patient health education was consistently highlighted across interviews. Healthcare professionals emphasized that education could empower patients by increasing their understanding of illnesses and promoting active participation in their long-term well-being.

*“Promoting acceptance of illness is a big subject*,* as is creating an understanding of illness. I can only do that to a limited extent when it comes to certain illnesses that I’m not very familiar with*,* but it’s also about illness perception*,* understanding illness*,* accepting that it’s part of me and that it’s not just something that defines me.”* (Psychotherapist, Germany).

Educating patients was noted to help reduce stigma and guide them toward suitable support while being cautious about self-diagnosis risks. Clear communication about the availability and use of existing resources was seen as crucial, along with the need for better promotion of these resources and easier access to information. In the Netherlands, reliable online information was reported to already exist, but there was a call for greater attention to these resources and the development of better prevention strategies. In Italy, the situation was perceived as much more restricted with very little adequate healthcare education on PSS. Promoting health literacy was also considered an important component of effective health education.

## Discussion

This study explored healthcare professionals’ perceptions of how European healthcare systems influence the course of symptoms in patients with PSS. The results indicate that the interaction of structural and interpersonal factors of the healthcare systems influences the course of PSS symptoms. Healthcare professionals mostly focused on systemic barriers, highlighting issues such as limited consultation times apparent throughout the healthcare system and barriers affecting specific stages of the patient care trajectory. There was a key split between countries on perceived barriers. These were barriers related to the structural framework of the system, like the organization of care pathways or insurance coverage in Germany and the Netherlands, and issues of access and trust of the system in Italy and Poland. Key improvements needed at the systemic level included sufficient reimbursement and eligibility of PSS for treatments, the establishment of care pathways for collaborative care, and opportunities for sufficient consultation times. Improvements identified at the interpersonal level included improvement in the professional-patient relationships. Linked to both was the need for improved education for healthcare professionals through formal curricula and continuous professional development opportunities, as well as increased educational initiatives and improved patient education resource accessibility.

### Comparison with the literature

The systemic barriers identified by healthcare professionals in our study align with previous studies [9, 18, 25]. These studies reiterate the barriers of insufficient consultation time, lack of clear care pathways, referral issues, and inadequate interdisciplinary collaboration. The many barriers mentioned highlight the complexity of challenges patients and professionals face in managing PSS and the heterogeneity between the different healthcare systems. Our results further build on findings of a recent European-wide survey [18], offering deeper insights into the systemic challenges encountered by healthcare professionals across the same four countries.

The present study also demonstrated differences across countries, consistent with previous findings [11, 18]. German and Dutch healthcare professionals focused more on aspects related to the content or organization of care, while Italian and Polish healthcare professionals highlighted concerns about accessibility to care and lack of clear treatment pathways. This supports the complex interplay between policy differences, resources, and cultural factors shaping PSS management [18]. Therefore, local needs and context of healthcare professionals and organizations should be considered to guide future research and policymakers in development of tailored implementation strategies for PSS improvement [26].

In our study, there was a consensus that GPs should act as care coordinators, guiding patients, and having an overview of their care. Healthcare professionals highlighted that the absence of such coordinating figure in the care pathway can lead to patients being trapped in repetitive treatment cycles or undergoing unnecessary consultations. Even in the Netherlands, where GPs serve as gatekeepers [27], it was noted that gatekeeping alone does not equal effective coordination. Previous studies have shown that patients and healthcare professionals often prefer GPs as central figures in care coordination [28–30], valuing the continuity and consistency they can provide. However, this role is not always consistently implemented across healthcare settings [30]. Our findings support these perspectives and emphasize the need for stronger support structures to enable GPs to fulfill this role effectively, including improved communication between primary and secondary care [30, 31].

Another key common finding was the varying levels of knowledge and experience among healthcare professionals, the impact this has on patients’ symptoms, and the provision of effective care. The lack of proper training for healthcare professionals aligns with previous literature, demonstrating that current curricula worldwide are insufficient in preparing professionals to communicate effectively with patients about their symptoms [32, 33]. This gap in education contributes to healthcare professionals’ perceived lack of competence [10] and stigma toward patients with PSS [34, 35], which in turn can affect patient care by healthcare professionals over-investigating or wanting to avoid patients with PSS [10]. However, it was also noted in our interviews that this lack of knowledge, while creating a sense of hopelessness, fostered motivation for self-learning among some healthcare professionals. This highlights the importance of experience, as practitioners often learn from working with patients when formal knowledge is lacking [36, 37]. Therefore, enhancing educational programs and providing continuous professional development can bridge this gap.

### Strengths and limitations

To our knowledge, this is the first known study to explore how healthcare factors across multiple countries can shape the course of PSS. A key strength is the qualitative analysis conducted in the native languages of participants, which prevented the loss of nuanced, language-specific, and culture-specific content [21]. This study builds on earlier quantitative approaches [9, 18, 25] to identifying barriers in healthcare systems, and utilizes open and in-depth interviews to draw attention to specific areas of needed improvements. While we ensured diversity in the sample by healthcare professionals, a potential limitation of the study is that these participants were initially contacted through existing research networks. Therefore, they were self-selecting and likely to have an active interest in diagnosing and managing PSS. While these perspectives might not reflect the full spectrum of healthcare professionals’ experiences, we were not aiming to describe the system in full detail but to gain detailed insights. Additionally, we included participants representing different specialties in each country and used a topic guide to ensure a consistent approach to the interviews. We were careful to frame the results as reflecting the views of the participating professionals rather than generalizing to national or professional populations. Finally, although the sample size was relatively small in each country, the coding process was extensive and iterative, involving a year-long team-based analysis with regular discussions. Toward the end of this process, no new codes emerged, indicating that thematic saturation was reached at the coding level.

### Future directions and implications

One of the implications of better supporting GP-level coordination of care might be to improve the use of specific codes to record patients’ symptoms, enabling earlier identification of people with PSS [38]. Furthermore, PSS care consumes significant healthcare resources, and GPs already struggle with limited time and increasing workloads [9, 39]. Therefore, advocating for GPs to have more time with patients with PSS is essential for better care coordination, avoiding unnecessary referrals, and reducing the waste of limited healthcare resources. Effective practices mentioned in this study include the Dutch advocacy campaign “Meer tijd voor patiënt” (“More time for patient”), which extends consultation times for complex cases, and in Germany, where GPs are better compensated for longer consultations through specific billing codes within the statutory health insurance system. Both initiatives aim to provide more comprehensive care by enabling healthcare professionals to delve deeper into the various factors affecting a patient’s symptoms and experiences. Future research should continue to evaluate the impact of these measures. To facilitate implementation of such practices, structural changes such as policy reforms and adjustments in the healthcare reimbursement models are necessary. Policy-makers need to take these approaches into account and develop a more flexible and adaptable service that allows GPs to allocate more time for patients with PSS, while effectively coordinating their care.

Healthcare professionals across all countries have reiterated the need for interdisciplinary collaboration and multidisciplinary care, consistent with previous findings [18, 31, 40, 41]. Though emphasizing different needs in multidisciplinary care, it is essential to consider the local context of healthcare systems. In Germany and the Netherlands, multidisciplinary care is available and supported by existing guidelines [42, 43], yet could benefit from further enhancement. Conversely, there is a significant need to implement such integrated care in Italy and Poland, as they lack guidelines emphasizing multidisciplinary care. Future research should focus on developing and evaluating such guidelines. Policymakers should prioritize supportive frameworks encouraging interdisciplinary collaboration and resource allocation for such initiatives. The introduction of “Blankoverordnung” (“Blank/open prescription”) in Germany marks a significant policy innovation, by allowing therapists to determine treatment specifics flexibly, such as duration and methods, and thereafter enhancing multidisciplinary care by providing more personalized and adaptive treatment options. However, further studies are needed to understand how patients utilize the healthcare system under this new regulation and its impact on multidisciplinary care. Additionally, further health economic studies should investigate whether collaborative care reduce healthcare costs, as previous studies suggest it might not be cost-effective [44]. When adopting practices from other countries, it is essential to ensure alignment with the local healthcare context. The EU’s approach to rare diseases, which involves establishing extensive networks for research, development, and dissemination, along with implementing national plans, shows the effectiveness of coordinated efforts that can be adapted by individual countries [45]. This model could similarly benefit PSS care by providing coordinated European resources adaptable to local contexts.

To address insufficient knowledge and training, PSS should be incorporated into formal education curricula, and joint training sessions should include all relevant professionals, ensuring they are well-versed in the biopsychosocial model of care [35, 46]. Measures are already being taken in this direction. One example is a study, which developed a communication intervention explaining PSS to patients to support self-management, which led to sustained improvement of symptoms [47]. Additionally, a successful communication initiative in the Netherlands enhanced provider-patient interactions using a blended learning approach [48]. Both programs focus on improving empathetic communication thorough symptom exploration, shared understanding, and tangible explanations. While significant steps have been made, there is a need to improve outreach and implementation. Continuous professional development and encouraging ongoing interactions between professionals from different countries by implementing regular knowledge exchange programs and international workshops can lead to the sharing of best practices and innovative approaches.

## Conclusion

This study provides valuable insights into healthcare professionals’ perceptions of the care situation for people with PSS, highlighting barriers and facilitators that can ultimately shape the symptom course and patients’ experiences within the healthcare system. It highlights that despite improving expertise, systemic barriers often prevent professionals from utilizing it effectively. Addressing these challenges requires a comprehensive and context-specific approach. Key strategies include recognizing and responding to country-specific needs, enhancing healthcare professionals’ training, alleviating time constraints, and improving GP care coordination. Healthcare systems should also recognize that PSS can develop into a chronic condition requiring long-term treatment, to ensure that patients are eligible for necessary treatments that are not currently covered by insurance. Further research is needed to better understand how healthcare systems influence the symptom course of PSS. Implementing these steps has the potential to significantly improve the quality of care and patient outcomes for people with PSS.

## Supplementary Information

Supplementary Material 1.

Supplementary Material 2.

## Data Availability

Transcripts of the interviews gathered and analyzed during the current study are not publicly available due to the potential inclusion of identifiable information, which could compromise the privacy and confidentiality of the participants. Registration and further study materials can be found on the OSF platform: https://osf.io/j63s4/.

## Abbreviations

PSS: Persistent Somatic Symptoms
GP: General Practitioner
ETUDE: Encompassing Training in fUnctional Disorders across Europe
OSF: Open Science Framework
EURONET-SOMA: European Research Network to improve Diagnosis, Treatment and Care for Patients with Persistent Somatic Symptoms
MAXQDA: MAX Qualitative Data Analysis
MRI: Magnetic resonance imaging
MVZ: Medizinische Versorgungszentren (Medical Care Centers in Germany)
