# Supplementary material for: Healthcare professionals’ views on healthcare-related factors influencing symptom course in persistent somatic symptoms: a qualitative study of four European countries
Source: BMC Health Serv Res. 2025 Jun 11;25:823. doi: 10.1186/s12913-025-12986-1 (PMC12153185; doi:10.1186/s12913-025-12986-1)
Supplement: Supplementary file 1 — Supplementary Material 1. [file 12913_2025_12986_MOESM1_ESM.pdf]

Supplementary Material 1

Professional Background of Study Group

	Sex	Credentials	Occupation	Experience and training	Country coded
**AKM***	Female	PhD student	Junior researcher		Netherlands, Poland
**BMF**	Male	PhD student	Junior researcher	Prior experience with qualitative studies	Netherlands
**TP**	Female	PhD student	Junior researcher		Italy
**AW***	Female	PhD	Licensed psychotherapist, postdoctoral researcher	Prior experience with qualitative studies	Germany
**KFP**	Female	PhD student	Psychologist and research assistant		Poland
**BL**	Male	MD	Project supervisor	Prior experience with qualitative studies	Germany
**JR***	Female	Professor	Project supervisor	Prior experience with qualitative studies	
**FC**	Female	Professor	Project supervisor		

Note. * = conducted interviews.
