# Supplementary material for: Healthcare professionals’ views on healthcare-related factors influencing symptom course in persistent somatic symptoms: a qualitative study of four European countries
Source: BMC Health Serv Res. 2025 Jun 11;25:823. doi: 10.1186/s12913-025-12986-1 (PMC12153185; doi:10.1186/s12913-025-12986-1)
Supplement: Supplementary file 2 — Supplementary Material 2. [file 12913_2025_12986_MOESM2_ESM.pdf]

Supplementary Material 2. *Thematic Codebook - Codes for Each Theme, Code Descriptions, and Anchor Examples.*

Systemic barriers

First contact barriers

Factors that hinder the delivery of good quality care for patients with PSS during their initial contact, usually in the primary care setting.

"There's also this stereotype perpetuated that GPs somehow don't have the competence and qualifications to treat psychosomatic symptoms or symptoms under the somatic form. ... patients don't trust GPs in this area." (Psychiatrist, Poland)

Lack of care pathways

Absence or inadequacy of structured treatment pathways for patients with PSS.

"The problem is always the usual one, i.e. it revolves around the fact that since there are no dedicated pathways the accessibility is poor and so they are/ the risk is that there is risk/ what often happens is that these patients um [reflecting] *wander a bit aimlessly, if you can call it that, and so they don't find their place* [in terms of specialized care], *they don't come i.e./ or the intake is done very late."* (Internist, Italy)

Issues with referral practices

Challenges related to the referral process for patients with PSS, e.g., limited familiarity with PSS in primary care, varying level of interest, limitations in referral options.

"...sometimes, we also see very brief referrals like, 'Oh, someone has pain, please help.' And then, um, you don't know what was said there ... It's almost more of an issue with the referrer. 'Please help,' you think, well, for the patient or for, um, for you?" (Rehabilitation Specialist, the Netherlands)

Lack of specialized professionals

Scarcity of healthcare professionals or their will to treat patients with PSS.

"Nowadays, patients are sometimes sent to me specifically because there are not so many specialized options for people with chronic complaints, they don't get as much support in the practice, in the outpatient clinic outside, so to speak." (Gynecologist, Germany)

Long waiting times

Perspectives of healthcare professionals on the delays patients with PSS encounter when attempting to access healthcare services, across various healthcare disciplines.

".. And they [the patients] *wait like that for a couple of months, half a year, where there's just such a progression of the illness and sometimes it's too late to reverse the cause."* (Psychiatrist, Poland)

Lack of specialized facilities

Absence of dedicated clinical units, specialized clinics, or facilitates focused on treating PSS.

"I believe that in rural regions, there is significant under-provision not only in the hospital but also in the outpatient sector for somatic issues. And for psychosomatic competence or linked psychosomatic competence, one could certainly create a large shortage map." (Gynecologist, Germany)

Note. PSS = Persistent Somatic Symptoms.

Systemic barriers

Lack of consultation time

Barriers and challenges healthcare professionals face in relation to the amount of time allotted for patient consultations.

"I think the problem, as I understand it from patients, is often a lack of time. When patients actually want to say a lot more than the doctor is perhaps signaling that they want to listen. We have time pressure in general practice. Especially when there are a lot of acute patients in addition to the appointment consultation hours, especially now with corona and colds." (GP, Germany)

Out-of-pocket expenses

Financial burdens patients encounter when seeking treatment for PSS, including costs associated with supplementary insurance, private clinics, and out-of-pocket payments.

"There's a certain difficulty, certain roughness, discomfort with public healthcare, that if everything went smoother there, maybe patients would think less about financing treatment from their own funds." (Psychiatrist, Poland)

System pressures

Systemic challenges, which affect the delivery of care, such as high patient loads, excessive administrative burdens, rigid time management structures.

"I think the system causes that an overworked doctor, nurse, social worker, psychologist, psychiatrist, anyone who is overwhelmed with formalities, above all, not even by the patients themselves, but by the formalities, to be overwhelmed and unable to approach the patient with genuine interest because they themselves are simply tired, exhausted." (Physiotherapist, Poland)

Resource barriers (HCPs)

Views on challenges faced by healthcare professionals in delivering comprehensive care due to limitations in financial, staffing, institutional support, and geographical factors.

"There is also a problem with the medical staff. In my department, only one nurse is not retired. Most of the individuals are those who have already reached retirement age and are working to supplement their income, but they will not work forever either, so in five years, for example, I estimate that there will be a very significant staff shortage. So, even if we allocate more money to the system, for instance, it won't completely solve the problem." (Rehabilitation Specialist, Poland)

Insurance coverage barriers

Barriers related to insurance coverage or reimbursement schemes, which affect the treatment and management of PSS.

"These are disorders that pay little [to us doctors] *compared to the many diagnostic investigations."* (Internist, Italy)

Resource barriers & disparities (patients)

Healthcare professionals' views on resource barriers patients with PSS encounter in accessing healthcare services, e.g., financial difficulties, lack of support services, and socioeconomic disparities.

"There is definitely a big difference between people who have the opportunity to afford medical services themselves and those who have no money. So there is a social gap when it comes to these things." (Gynecologist, Germany)

Note. PSS = Persistent Somatic Symptoms; HCPs = Healthcare Professionals.

Systemic barriers

Timely diagnosis barriers

Challenges and barriers identified by healthcare professionals that hinder the timely and accurate diagnosis of patients with PSS, e.g., communication problems, diagnostic approaches, systemic factors, provider knowledge.

"It's easier to identify a heart attack than to identify a persistent somatic symptom disorder. Somehow, everything that is more blurred, less precise, requires much more attention and involvement. That is, if you don't feel motivated to look for it, you'll never find it [laughs].*"* (GP, Italy)

Early and adequate treatment barriers

Obstacles identified by healthcare professionals that hinder the timely and effective provision of treatment for patients with PSS.

"But our treatment frameworks are still focused on musculoskeletal complaints, while our knowledge is increasingly about, you know, the functional level or the brain and the stress system. And with that, we see that we could actually treat people with general SOLK [medically unexplained symptoms] *very well in these settings because they are dealing with the underlying patterns. But because of our own treatment frameworks and also because of the Health Insurance Act, we are not allowed to treat those patients."* (Rehabilitation Specialist, the Netherlands)

Diagnostic labels (influence on care)

Healthcare professionals' views on ways in which diagnostic labels affect care delivery and patient outcomes.

"The problem lies again with the healthcare system. There is no label provided in the remedy prescription for physiotherapy. That means, it always states something like cervical spine syndrome or lumbar spine syndrome or um... (4 sec.) heart failure, anyway. There must be a diagnosis according to this indication catalog. And there's a deficiency!... For physiotherapy, in the... when the indication must be stated, there are such designations, then there must be something written there that fits into the remedy prescription." (Physiotherapist, Germany)

Delayed or inadequate treatment or system failure to prevent chronicity

Situations in which healthcare professionals neglect to treat PSS right away, which can lead to inadequate or delayed treatment and possibly chronicity, made worse by excessively invasive diagnostic or therapeutic methods.

"But the specialist generally tends to rule out the slightest suspicion of organic nature by the most invasive means." (GP, Italy)

Lack of specific diagnostic procedures

Challenges and limitations related to the diagnostic procedures available for identifying PSS.

"Another obstacle could also be the types of diagnostic tools that are used. So if we only use the DSM we will have a lot to read. You probably also have to consider other tools, I don't know the (DCPR?) for example, other tools." (Psychotherapist, Italy)

Note. PSS = Persistent Somatic Symptoms.

Systemic facilitators

First contact facilitators

Factors that facilitate the delivery of good quality care for patients with PSS during their initial contact, usually in the primary care setting.

"If you have a general practitioner who has more affinity with PSS and dares to discuss it with someone, that can definitely help." (Physiotherapist, the Netherlands)

Insurance coverage facilitators

Views on factors within the healthcare system that facilitate coverage for patients.

"We have a healthcare system that is recognized, from this point of view, as a constitutional right." (GP, Italy)

Accessibility of care facilitators

Mention of factors related to the ease of access to healthcare services or resources that facilitate care.

"So, when it becomes a recognized service even within these types of pathways, then a network can be created at the local/regional, hospital, or healthcare system level. And so, there can be greater accessibility to the treatments, without having to turn perhaps to professionals who do not communicate with each other." (Nutritionist, Italy)

Consultation time facilitators

Views on factors within healthcare system that facilitate time dedicate for patient consultations.

"It's easier in the office because there I organize my work myself, so I can do it as I want, so here, if I plan too short a time, it's my mistake or oversight. Here I can think about it." (Psychiatrist, Poland)

Desired change

Perspectives from healthcare professionals on areas that they believe need improvement or reform within the system to improve outcomes related to PSS.

"Hm [in the affirmative] *So now, in terms of physiotherapy, I would like the initial contact to be extended. When we get a prescription for a remedy. It says yes/ everything is regulated. Six times so and so on it. And basically you'd have maybe 15 to 20 minutes and then you have to, we have to listen/ so anamnesis, then goal, actually talk about goals and so on. And also do a physical examination. That's impossible."* (Psychotherapist, Germany)

Effective practices

Healthcare professionals' expressions of established practices or initiates within healthcare that are effective for patients with PSS.

"Well, we've actually had a lot more time recently, but that's because we've extended from ten to 15 minutes. So that is a kind of, uh, project. It's called more time for the patient." (GP, the Netherlands)

Ideas and solutions

Mentions of specific ideas or solutions aimed at improving the management of treatment of PSS within the healthcare system.

"And the alternative is MVZ [Medical Care Center] *and we do have a structure for that. I would actually find it better for the future to offer that within the framework of an MVZ. In urology, it's already being done like that, and then, um, someone with an advanced training can also bill for psychosomatic codes and so on."* (Gynecologist, Germany)

Note. PSS = Persistent Somatic Symptoms.

Multidisciplinary care

Coordination of care needs

Expressions of the need for a designated coordinator or coordinating figure (e.g., GP) to oversee and guide patient care processes and enhance practitioner collaboration and communication.

"…there's a bit of a lack of coordination like the one we talked about at the beginning. That would be useful… there's a bit of a lack of this space for contact, like for example joint consultations, joint treatment in the hospital." (Psychiatrist, Poland)

Coordination of care facilitators

Facilitators and practices enabling effective coordination of care among healthcare providers, including communication, collaboration, and supportive roles within treatment pathways.

"Well, what we do is also inform the general practitioner again about what we are doing or planning with the patient. So, at least he [the GP] *is then informed."* (Psychotherapist, the Netherlands)

Coordination of care barriers

Challenges and barriers that make it difficult to coordinate care for patients with PSS, e.g., a lack of financial incentives, of coordinating figure, of affinity; patients navigating the system on their own; structural aspects of care.

"And I would say, in our healthcare system, um, there's a big, um, need for a communication/ so there should be communication platforms created. That is this sectoral. That is what I would explain to someone from abroad. That we have the inpatient here and there we have the outpatient. They do not even link up, do they? It is not even promoted, paid for. I think that is increasing now, but I already find that a hitch. And also, that there's no/ still no digital patient record." (Physiotherapist, Germany)

Integration of psychological care

Healthcare professionals' recognition of the necessity and importance of integrating psychological care in treatment plans for patients with PSS.

"However, there is still a great lack of psychological support, and this is known to have a significant impact on the feelings themselves, the sensation of pain." (Rehabilitation Specialist, Poland)

Interdisciplinary collaboration barriers

Obstacles and challenges encountered by healthcare professionals when attempting to collaborate across disciplines to provide care for patients with PSS.

"We have a lot of psychology services that are difficult to reach, psychiatry equally difficult. […] If you think you can request a consult with them, as you would request a consult for a cardiovascular or cardiac condition from a cardiology service, this is not possible. There is no such thing g/ or at least in our reality it does not exist." (GP, Italy)

Need for multidisciplinarity

Healthcare professionals' recognition and advocacy of the implementation of collaborative and multidisciplinary care approaches to meet the needs of patients with PSS.

"…And the facilitators, it can be a multidisciplinary team. So the interaction between different professionals who are trained and obviously also possibly a dedicated treatment trajectory um [reflecting] *with consultants; when the trajectory exists they can be called to be consulted, perhaps by the doctor who, who may have this hunch, but not the experience and skills to recognize it promptly."* (Nutritionist, Italy)

Note. PSS = Persistent Somatic Symptoms; GP = General Practitioner.

HCPs' knowledge and experience

Training/ formal education

Extent to which the topic of PSS was covered in the formal education or training of healthcare professionals.

"Sure, in theory, it was covered. From today's perspective or from the perspective of the early years of work, I found that it wasn't enough to … get a good handle on dealing with the patients, and also to, well, to get a good approach there. I didn't feel well-trained in that regard." (Psychotherapist, Germany)

Learning on the job/ work experience

Gained experience and knowledge about PSS through practical work encounters and recurring patient interactions.

"… there hasn't been, let's say, a very thorough training. This rather comes from post-graduate training. Through research activities or clinical fieldwork and participation in international conferences." (Psychotherapist, Italy)

Attitudes about patients

Healthcare professionals' negative perceptions and behaviors towards patients with PSS.

"They are also more complex and time-consuming to begin with, and they might get on your nerves at some point because they take resources away from others." (Physiotherapist, Germany)

Self-directed learning

Independent learning activities to gain knowledge and skills related to the management of patients with PSS (e.g., attending conferences or workshops, reading relevant literature, seeking out guidance from experienced colleagues).

"At work, I was helpless in the face of these disorders, the symptoms, and the patients. I lacked a language for communicating with patients and I lacked an idea of how to treat this disorder. So one could say that I have educated myself in this area." (Psychiatrist, Poland)

Knowledge about PSS

Perceptions on the general state of knowledge of healthcare professionals about PSS in the respective countries.

"With my fellow specialist physicians in mental healthcare, we have peer supervision, which often revolves around PSS by the way… to raise awareness of PSS more. So, I suspect that in other regions as well, it's just much more openly discussed than before." (GP, the Netherlands)

Note. PSS = Persistent Somatic Symptoms.

Relationship and communication

Professional-patient relationship

Healthcare professionals' perspectives on the importance, challenges, and elements of their relationship with the patient concerning PSS.

"And if sometimes they end up with a specialist ... it turns out that in this relationship they [the specialist] *is a very cold person, adopting an attitude of such lack of interest in the patient, then, I think, for this patient it's probably the worst."* (Physiotherapist, Poland)

Timely diagnosis facilitators

Factors identified by healthcare professionals that facilitate timely diagnosis in PSS patient care.

"Hm [thoughtfully] *more like a clearing office/center. So where you, um, perhaps, as a family doctor, you first work through a docket like that with, um, special areas with special diagnostics, what is still missing. And either you have the confidence to classify it yourself or you, as I said, have a psychosomatic outpatient clinic where a diagnosis can then perhaps be made based on the previous findings."* (GP, Germany)

Adequate care

Views of healthcare professionals as to what would constitute adequate care for patients with PSS.

"...it's not all the same, so simply personalized..." (Physiotherapist, the Netherlands)

Health education/promotion

Recognition of the importance of education of patients about health-related issues to empower them and enhance their health outcomes.

"And as for the topic of health literacy, well, practically not at all for the population." (Physiotherapist, Germany)

Holistic vs. particular viewpoint

Healthcare professionals' views on the importance of adopting a holistic rather than a biomedical approach for managing PSS.

"What you often see is that, uh, so, besides having physical complaints, someone encounters difficulties in many areas of life. And yes, I think it's good if you can at least map this out in an early stage or in an early phase." (GP, the Netherlands)

Note. PSS = Persistent Somatic Symptoms.
